# One-Step Enrichment of Intact Glycopeptides From Glycoengineered Chinese Hamster Ovary Cells

**DOI:** 10.3389/fchem.2020.00240

**Published:** 2020-04-17

**Authors:** Ganglong Yang, Naseruddin Höti, Shao-Yung Chen, Yangying Zhou, Qiong Wang, Michael Betenbaugh, Hui Zhang

**Affiliations:** ^1^Department of Pathology, Johns Hopkins University, Baltimore, MD, United States; ^2^Chemical and Biomolecular Engineering, Johns Hopkins University, Baltimore, MD, United States

**Keywords:** intact glycopeptides, glycoengineered CHO cells, one-step enrichment, *FUT8* knockout, mass spectrometry

## Abstract

Recently, the glycoproteomic analysis of intact glycopeptides has emerged as an effective approach to decipher the glycan modifications of glycoproteins at the site-specific level. A rapid method to enrich intact glycopeptides is essential for the analysis of glycoproteins, especially for biopharmaceutical proteins. In this study, we established a one-step method for the rapid capture of intact glycopeptides for analysis by mass spectrometry. Compared to the conventional sequential enrichment method, the one-step intact glycopeptide enrichment method reduced the sample preparation time and improved the detection of intact glycopeptides with long sequences or non-polar amino acids. Moreover, an increased number of glycosite-containing peptides was identified by the one-step method compared with the sequential method. When we applied this method to the glycoproteomic analysis of glycoengineered Chinese hamster ovary (CHO)-K1 cells with α1,6-fucosyltransferase (*FUT8*) knockout, the results showed that the knockout of *FUT8* altered the overall glycosylation profile of CHO-K1 cells with the elimination of core fucosylation and together with increases in high-mannose and sialylated N-glycans. Interestingly, the knockout of the *FUT8* also appeared to regulate the expression of glycoproteins involved in several functions and pathways in CHO-K1 cells, such as the down-regulation of an intracellular lectin LMAN2 showing cellular adaptation to the alterations in *FUT8* knockout cells. These findings indicate that the site-specific characterization of glycoproteins from glycoengineered CHO-K1 cells can be achieved rapidly using the one-step intact glycopeptide enrichment method, which could provide insights for bio-analysts and biotechnologists to better tailor therapeutic drugs.

## Introduction

It has been challenging to interpret the heterogeneity of glycoproteins, which includes protein sequence, glycosite information, glycan structures at each glycosite, and overall and individual glycan occupancy at specific sites (Liu et al., 2017). Recently, the mass spectrometric analysis of intact glycopeptides has emerged as a promising strategy to analyze glycoproteins for glycan modifications at site- and structure-specific levels (Sun et al., 2016; Yang et al., 2018; Xiao and Tian, 2019). However, the major limitation for this approach is the low detectability of intact glycopeptides in a complex mixture, which are suppressed by non-glycosylated peptides (Shajahan et al., 2017). Therefore, a capture method for intact glycopeptides before mass spectrometry analysis is highly desirable.

Several approaches, including the immobilization of glycopeptides using hydrazide chemistry (Zhang et al., 2003; Nilsson et al., 2009), lectin enrichment (Kaji et al., 2003; Yang et al., 2013), hydrophilic interaction chromatography (HILIC) (Wada et al., 2004; Sun et al., 2016), and anion exchange (Yang et al., 2017), are reported for glycopeptide capture. Among them, HILIC is commonly used for intact glycopeptide enrichment. Typically, the preparation of intact glycopeptides using HILIC involves several steps. Briefly, the proteins are digested to peptides by protease(s), followed by peptides clean-up by hydrophobic chromatography, drying, and then reconstitution in the organic solvent for glycopeptide enrichment using hydrophilic enrichment.

**Figure 1:**
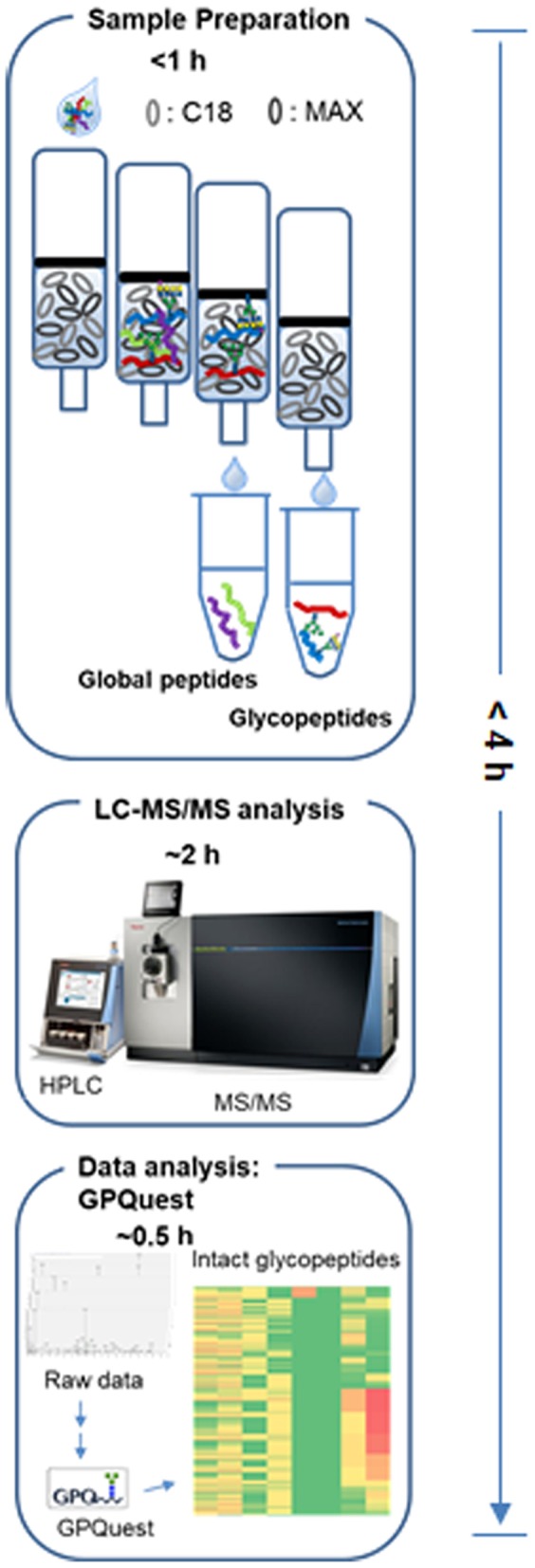
The workflow of one-step enrichment and analysis of intact glycopeptides.

In this study, we incorporated the hydrophobic and the hydrophilic chemistries into a one-step column for enrichment of glycopeptides (Figure 1). The one-step method was applied to the capture of intact glycopeptides from glycoengineered Chinese hamster ovary (CHO)-K1 cells with α1,6-fucosyltransferase (*FUT8*) knockout. The captured intact glycopeptides from glycoengineered and wild-type CHO-K1 cells were analyzed by mass spectrometry. The results showed that, in addition to eliminating core fucosylation, the *FUT8* knockout altered the overall glycosylation profile of CHO-K1 cells with increases in high-mannose and sialylated N-glycans, as well as the expression of proteins that were involved in several functions and pathways. The results showed that the rapid capture and the analysis of site-specific characterization of glycoproteins from glycoengineered CHO-K1 cells can be used for the fast characterization of glycoproteins expressed from glycoengineered CHO cells to better tailor therapeutic drugs.

## Materials and Methods

### Protein Digestion

Proteins (500 μg) from wild-type CHO-K1 and *FUT8* knockout (KO) CHO-K1 cells were denatured in 8 M urea/1 M NH_4_HCO_3_ buffer, reduced with 10 mM TCEP at 37°C for 1 h and alkylated with 15 mM iodoacetamide at room temperature for 30 min in the dark. The solutions were diluted 8-fold with ddH_2_O. Then, the sequencing-grade trypsin (protein: enzyme, 40:1, w/w; Promega, Madison, WI) was added to the samples and incubated at 37°C for 30 min to overnight. For the traditional approach, the peptides were acidified by acetic acid with pH <3. The samples were centrifuged at 13,000 g for 10 min, and the supernatant was cleaned by C18 solid-phase extraction. The peptides were eluted from the C18 column in 60% acetonitrile (ACN)/0.1% TFA, and the peptide concentrations were measured by bicinchoninic acid protein assay. For the one-step method, the peptides were acidified like the traditional approach and then applied to the conditioned one-step columns.

### Preparation of Columns for the One-Step Enrichment Method

For the mixed columns, 30 mg mixed anion exchange (MAX) beads and 50 mg C18 beads were suspended in 1 ml ACN and mixed homogeneously in a tube. Then, 100 μl of the mixture was packed into a column with filters at the bottom and at the top and followed with a single wash using 1 ml ACN. For the stacked columns, the MAX beads (30 mg, Waters) were packed into a column with a filter at the bottom first and washed with 1 ml ACN three times. Subsequently, the C18 beads (50 mg) in ACN were packed onto the top of the MAX beads followed with a filter on the top and washed once with 1 ml ACN.

### Enrichment of Intact Glycopeptides

The columns were sequentially conditioned in ACN three times, 100 mM triethylammonium acetate three times, 95% ACN (v/v) with 1% TFA (v/v) five times, and finally 1% TFA(v/v) five times. The samples were loaded twice in 1% TFA (v/v). The columns were washed with 1% TFA four times. Then, the non-glycopeptides were eluted with 95% ACN (v/v) with 1% TFA (v/v) three times. Finally, the intact glycopeptides were eluted in 50% ACN (v/v) with 0.1% TFA (v/v) and dried in a speed-vac in preparation for mass spectrometry.

### Nano-LC–MS/MS Analysis and Data Analysis

The mass spectrometric method and data analysis are described in our previous publication with modification (Yang et al., 2018). In brief, the peptide samples in 3% ACN and 0.1% FA were analyzed with a Q-Exactive or Orbitrap Fusion Lumos Tribrid mass spectrometer (Thermo Fisher Scientific). The data were searched using glycopeptide analysis software GPQuest 2.0 (Toghi Eshghi et al., 2015; Hu et al., 2018), which was developed in-house. The results were filtered with false discovery rate (FDR) and glyco-peptide spectrum match (PSM) count number.

## Results and Discussion

### Rapid Capture of Intact Glycopeptides From Fetuin by the One-Step Enrichment Method

First, we investigated the performance of the one-step enrichment by two strategies for packing C18 and MAX particles into one cartridge. In one strategy, the C18 and the MAX beads were mixed homogeneously in acetonitrile and then packed into one column (mixed column), while in the other strategy, the C18 and the MAX beads were packed sequentially as a stacked C18 and MAX column (stacked column). Using the mixed column or stacked column, the IGPs were enriched from the mixture of peptides by the one-step method, in which the peptides were bound to C18 particles in the column, desalted by washing with 5% ACN, and eluted with 95% ACN. Then, the eluted IGPs directly bound to the MAX particles through hydrophobic interaction in the same column; the non-glycopeptides were washed away by 95% ACN. Then, the IGPs were eluted with 50% ACN from the one-step method. Meanwhile, these two one-step enrichment strategies were also compared with a traditional sequential method using two individual enrichment steps (sequential method) that we described in our previous studies (Yang et al., 2017, 2018), in which the peptides were prepared by hydrophobic chemistry using C18 column, eluted with 60% ACN. The eluted peptides were dried, and the IGPs were enriched by hydrophilic chemistry using a MAX column.

Next, we evaluated the one-step enrichment on its performance to enrich intact glycopeptides (IGPs) from bovine fetuin, which worked as a model protein in this study. Briefly, the bovine fetuin was first digested wit trypsin, and the tryptic peptides were enriched for IGPs by the one-step methods (mixed or stacked columns) and the traditional sequential glycopeptide enrichment method (sequential method), respectively. The peptides with and without glycopeptide enrichment were run on a Q-Exactive mass spectrometer using our previous IGP analysis parameters (Yang et al., 2018). The glycosite-containing database was comprised of three N-linked glycosite-containing peptides from bovine fetuin (P12763, up to two missed cleavage sites) (Table S1). For the N-glycans and O-glycans database, we performed the glycomics analysis of fetuin using the reported glycan permethylation method (Ciucanu and Costello, 2003) as well as using the N-glycans identified from our previous report using NGAG method (Sun et al., 2016). The glycosite-containing peptides and glycans generated databases containing 16 glycosite-containing peptides, 108 N-glycan structures (Figure S1 and Table S2 A), and 3 O-glycan structures (Figure S2). From our GPQuest search result, all the three known N-linked glycosites were identified by the global method without glycopeptide enrichment, the one-step method, and the sequential method for IGP enrichment (Table S2). From the heat maps of peptide-specific glycosylation distribution and global glycan distribution on fetuin, the global method without glycopeptide enrichment could only identify highly abundant glycosylated peptides on fetuin. The one-step methods were efficient for the enrichment of IGPs from all three glycosites, LCPDCPLLAPLN^#^DSR, RPTGEVYDIEIDTLETTCHVLDPTPLAN^#^CSVR, and VVHAVEVALATFNAESN^#^GSYLQLVEISR, while the IGPs from glycosite LCPDCPLLAPLN^#^DSR were preferentially enriched by the sequential method rather than the other two glycosites (Figure S3). The glycosite-specific IGPs carrying different glycans suggest that the sequential method is favorable for short glycosite-containing peptides, and the long glycosite-containing peptides get lost in the sequential method, possibly due to the challenging elution of long glycopeptides from C18 particles with 60% ACN elution solution, which is typically used to elute peptides from C18 particles. However, in the one-step method, the peptides were eluted from C18 particles with 95% ACN solution, which is more beneficial for long peptides with high hydrophobicity. Based on our results, 95% ACN solution could be used for the elution of peptides from C18 columns in the future. Collectively, the one-step method (both mixed and stacked columns) can efficiently enrich IGPs from the mixture of peptides. It is also worthy to mention that the one-step method requires less sample preparation process and time compared to conventional sample preparation methods.

### Rapid Enrichment of Intact Glycopeptides From *FUT8* Knockout CHO-K1 Cell by One-Step Enrichment Method

Furthermore, we compared the one-step and the sequential methods to analyze the glycosylation profile from glycoengineered CHO-K1 cells. The CHO cells are currently the primary production platform in biopharmaceutical manufacturing (Hossler et al., 2009). We evaluated the alterations of the N-linked glycosylation in CHO-K1 cells caused by *FUT8* knockout (*FUT8* KO). The core fucosylation-deficient cell line was generated by knocking out the *FUT8* gene from the CHO-K1 cells (Wang et al., 2018). The FUT8 (α1,6-fucosyltransferase) catalyzes the transfer of a fucose residue to position 6 of the innermost GlcNAc residue of N-linked oligosaccharide (core fucosylation) of a glycoprotein (Miyoshi et al., 1999). The removal of core fucosylation of pharmaceutical glycoproteins, especially for mAbs, has positive effects on their potency and efficacy, such as antibody-dependent cellular cytotoxicity (Chung et al., 2017).

**Figure 2:**
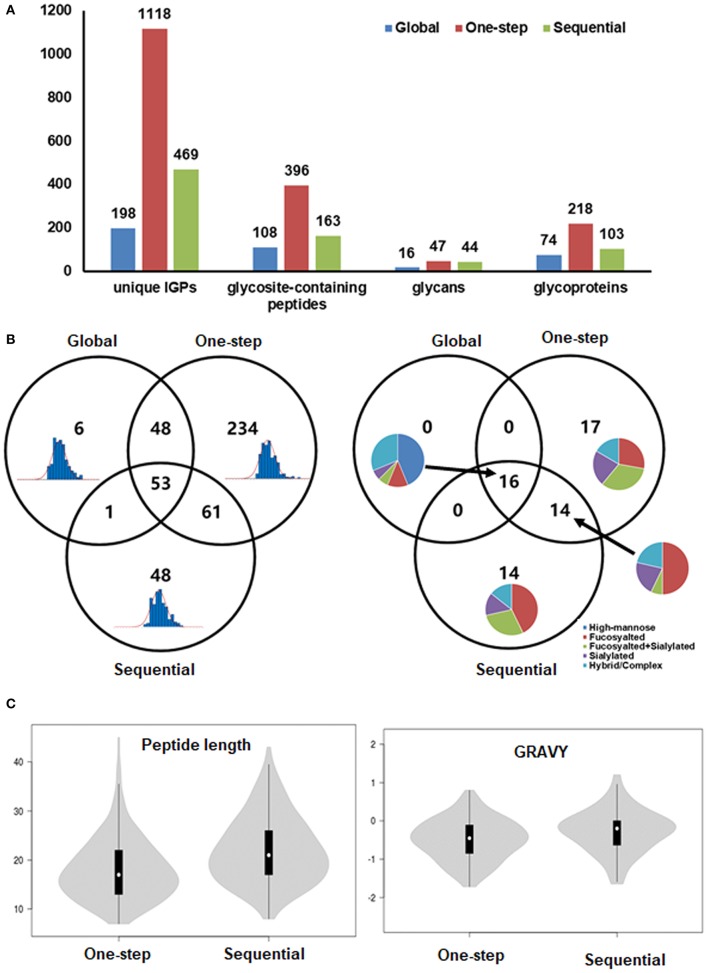
Enrichment of intact glycopeptides from *FUT8* KO CHO cells by one-step and sequential methods. **(A)** Identification and distribution of intact glycopeptides, glycosites, and glycoproteins identified from *FUT8* KO CHO cells by different methods. **(B)** Venn diagram of identified glycosites and their glycans by global, one-step, and sequential methods. **(C)** The length and grand average of hydropathy of identified glycopeptides enriched by one-step and sequential methods.

Because the stacked columns showed a similar performance as the mixed columns for the standard glycoproteins (Figure S3) but had an easier column preparation process, we then enriched all the glycopeptides from cells using the stacked columns for the following experiments. The peptides without and with enrichment were analyzed with an Orbitrap Fusion Lumos Tribrid mass spectrometer, of which 1,422 PSMs of IGPs, representing 1,118 unique IGPs, were detected by the one-step enrichment method, while 1,193 and 298 PSMs of IGPs, representing 469 and 198 unique IGPs, were detected by the sequential method or without enrichment (global method) (Figure 2A and Table S3). From the Venn diagram of the IGPs and the glycans identified by these three strategies, it showed that most of the IGPs and all the glycans identified by the global analysis were covered both by the one-step and the sequential methods. A total of 222 unique IGPs and 114 glycosite-containing peptides were overlapped by both the one-step and the sequential enrichment methods. The IGP number only identified by the one-step method (234) is 4.9-fold higher than the number identified exclusively by the sequential method (48). For the glycans from the IGPs, the high-mannose N-glycan were identified by all the three methods, and the sialylated glycans were most abundant in the one-step method (Figure 2B).

The results showed that the number of glycosite-containing peptides identified by the one-step method is 2.4-fold higher than that by the sequential method. However, the glycan numbers and the glycan types did not show much difference (47 vs. 44) between the two methods (Figure 2B). It indicated that the discrimination of the enrichment efficiency is caused by the difference of peptides. We next analyzed the property of the identified peptides, including the length and the grand average of hydropathy (GRAVY; indicating the hydrophobic or hydrophilic properties). It showed that there are no significant differences in the peptide length (including the distribution of the peptide length) and GRAVY, but like the fetuin result in Figure S3, some of the long peptides containing more than 40 amino acids were identified only by one-step methods. Some of the peptide GRAVYs from the sequential methods were higher than those of the one-step methods, which meant that the hydrophobicity of the peptide identified by the one-step methods is higher (Figure 2C).

### Analysis of Intact Glycopeptides From the Wild-Type and *FUT8* KO CHO-K1 Cells by the One-Step Method

**Figure 3:**
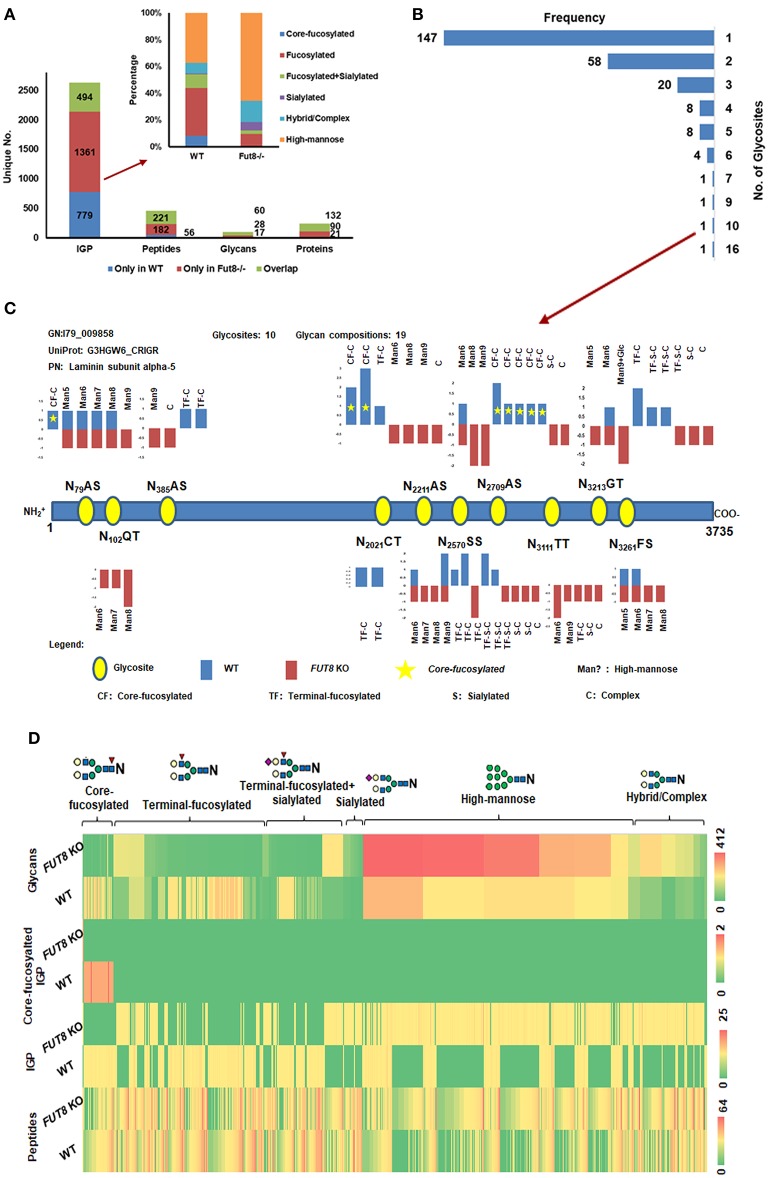
One-step enrichment of glycopeptides from WT and *FUT8* KO CHO-K1 cells. **(A)** Distribution of IGPs, glycosites, glycans, and glycoproteins identified from WT and *FUT8* KO CHO-K1 cells. **(B)** Glycosite frequency on the identified glycoproteins. **(C)** N-glycosylation of laminin subunit alpha-5 with 10 glycosites, including the structure type and relative abundance (peptide spectrum match) of glycans on each glycosite. **(D)** Heat map of glycosites, intact glycopeptides (IGPs), core-fucosyalted IGPs, and glycans of WT and *FUT8* KO CHO-K1 cells.

Given that the one-step column can efficiently enrich glycopeptides for glycoproteomic analysis of *FUT8* KO CHO-K1 cells, we next performed a comparison of the IGPs identified from wild-type (WT) and *FUT8* KO CHO-K1 cells using the one-step method. Peptides were prepared from cells cultured in three biological replicates and mixed together for glycopeptide enrichment using the one-step method. The enriched glycopeptides were analyzed twice using LC-MS/MS. We previously constructed a sample-specific glycopeptide database for the identification of N-linked glycosite-containing peptides using PNGase-F-released peptides (Yang et al., 2018). We applied the previous CHO-K1 cell glycomic results as glycan database (North et al., 2010). The databases used for the GPQuest search contain 57,653 potential glycosite-containing peptides and 343 glycan structure entries (Table S4). The spectra containing an oxonium ion (m/z 204.09) were chosen for further IGP searching. The results were filtered based on the following criteria: (1) FDR <1%, (2) ≥2 PSMs for each peptide were required, (3) all the PSMs should be annotated by at least one N-linked glycans, and (4) core Fuc fragment ions (peptide + HexNAcFuc) for core-fucosylated IGP were required. Using these filtering criteria, 2,634 unique IGPs were identified from WT and *FUT8* KO CHO-K1 cells, matching up to 459 glycosite-containing peptides, 243 glycoproteins, and 105 glycan compositions. For 2,634 unique IGPs, 1,273 IGPs were identified from WT CHO-K1 cells and 1,855 IGPs were identified from *FUT8* KO CHO-K1 cells (Table S5A). Additionally, we noticed that the ratio of overlap for IGPs between the WT and *FUT8* KO CHO-K1 cells was 18.8%, while the ratio of overlap for glycosite-containing peptides was 48.1%; for protein it was 54.3%, and for glycan it was 57.1% (Figure 3A). For the core fucosylation analysis, the FUT8 expression was significantly reduced, and only three PSMs of core-fucosylated IGPs were identified in *FUT8* KO CHO-K1 cells, while 127 PSMs of core-fucosylated IGPs were identified in the WT CHO-K1 cells. In addition, the overall proportion of the other glycosylated forms of IGPs was also remarkably changed, in which the proportion of fucosylated IGPs was significantly decreased; sialylated and high-mannose IGPs were increased in the *FUT8* KO CHO-K1 cells (Figure 3A).

For all the identified glycoproteins, we found that the glycosylation heterogeneity was also altered in *FUT8* KO CHO-K1 cells. In the 243 glycoproteins identified from the CHO-K1 cells using the one-step methods, over 60% of the glycoproteins were identified with only one glycosite, and about 3.3% of the glycoproteins were identified with more than five glycosites (Figure 3B). For example, from the hyperglycosylated protein laminin subunit alpha-5 with 10 identified glycosites, we noticed that the relative abundance was not changed after the *FUT8* KO, but the glycosylation at one glycosite (N_2021_CT) was lost and two neoglycosites (N_102_QT and N_3111_TT) were generated, which were mostly glycosylated by high-mannose N-glycans. For the core-fucosylated glycosites in WT cells, almost all the fucosylation were lost, and the high-mannose and complex glycans were increased (N_79_AS, N_2211_AS, and N_2709_AS). For the most microheterogeneity glycosite (N_2570_SS), the high-mannose and sialylated glycans were increased, which was similar to the tendency of glycosylation on all glycoproteins (Figure 3C).

Comparing *FUT8* KO with WT CHO-K1 cell samples, although the relative abundance of glycosite-containing peptides was similar to each other, the IGP and glycan profile has altered greatly. It displayed that the sialylated and most of the high-mannose IGPs and glycans were increased in the *FUT8* KO CHO-K1 cells compared to those in the WT CHO-K1 cells. Moreover, a heat map analysis of the relative abundance showed glycosite-containing peptides, IGPs, core-fucosylated IGPs, and glycans identified from the WT and *FUT8* KO CHO-K1 cells (Figure 3D). These results together demonstrated that the knockout of *FUT8* changed the glycosylation patterns of glycoproteins in CHO-K1 cells.

### Relative Abundance Alteration of Glycoprotein Expression in *FUT8* KO CHO-K1 Cells

**Figure 4:**
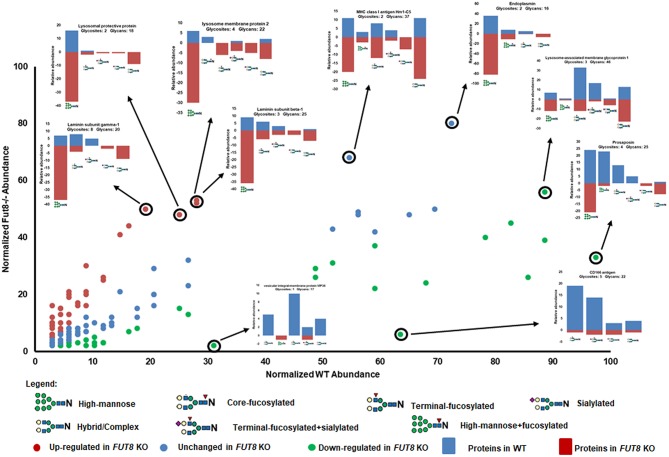
Relative abundance of glycoproteins between WT and FUT8 KO CHO-K1 cells. Glycosylation relative abundance of identified proteins between WT and FUT8 KO CHO-K1 cells, showing the correlation between the protein expression and their glycosylation abundance in WT and FUT8 KO CHO-K1 cells.

Interestingly, not only were the N-glycotypes altered but also the relative abundance of the glycoproteins was affected in *FUT8* KO CHO-K1 cells. We performed a comparative analysis of glycoproteins using label-free quantification methods based on spectral counting and normalized the data with the sum of spectra in each data set. A total of 40 glycoproteins were up-regulated (fold change >1.5) in the *FUT8* KO CHO-K1 cells. Contactin-associated protein 1 and voltage-dependent calcium channel subunit alpha-2/delta-1 were the top two among them (Figure S4). In these up-regulated glycoproteins, the diversity of glycans were also increased significantly, especially for the high-mannose and sialylated complex glycans. Regarding relative abundance, the fold change of glycans on some up-regulated glycoproteins is higher than the fold change of the glycoprotein expressions, like the high-mannose glycan on the protein laminin subunit gamma-1 (5.3 vs. 1.85) (Figure 4). On the other hand, a total of 31 glycoproteins were down-regulated (fold change <0.67) in the *FUT8* knockout CHO-K1 cells. Vesicular integral-membrane protein VIP36 (LMAN2) and CD166 antigen were the top two down-regulated glycoproteins. Intriguingly, we also noticed that the glycan diversity/abundance of these proteins was also sharply decreased as their relative glycoprotein abundance decreased. The relative abundance of some glycans was changed more rapidly than the abundance change to their corresponding glycoproteins (Figure 4 and Table S5B). For the glycoproteins with a consistent expression level between *FUT8* KO and WT CHO-K1 cells, the glycosylation of these proteins in the *FUT8* KO cells was also changed greatly, in which the high-mannose and sialylated complex glycans were increased compared to those in the WT CHO-K1 cells. Overall, the disruption of the *FUT8* gene not only caused the alteration of glycosylation profile on glycoproteins but also regulated the glycoprotein expression intracellularly.

Interestingly, the top four regulated glycoproteins are signal- and transmitting-related proteins. LMAN2 plays a role as an intracellular lectin in the early secretory pathway (Hara-Kuge et al., 1999). It interacts with GalNAc and high-mannose type glycans and is involved in the transport and sorting of glycoproteins (Yamashita et al., 1999). The down-regulation of LMAN2 could be a cellular adaptation to the increase in high-mannose glycoproteins. Furthermore, a total of 90 glycoproteins were exclusively expressed in the *FUT8* KO CHO-K1 cell but were not detected in the WT CHO-K1 cells, such as dipeptidyl peptidase 2, laminin subunit alpha-2, and transforming growth factor beta-1 (Table S5C). These three proteins play an important role in cell functions especially in terms of cell surface receptor binding (www.uniprot.org/uniprot/). About 63 glycoproteins of these 90 proteins were also identified by our previous proteomics analysis of the CHO-K1 cell (Baycin-Hizal et al., 2012). This discovery indicated that the suppression of α1,6-fucosyltransferase induced some non-glycosylated proteins to be glycosylated in the *FUT8* KO cells. To be specific, the majority (≥82%) of the intact glycopeptides identified from these 90 new glycoproteins contained high-mannose N-glycans, and only four glycoproteins did not contain any high-mannose N-glycan (Figure S5). This result indicates that these newly glycosylated proteins may not have complete glycosylation when they were processed through the Golgi apparatus.

In addition, the pathways containing significantly regulated glycoproteins were further analyzed by the Database for Annotation, Visualization, and Integrated Discovery software using the complete *Cricetulus griseus* proteome as background (Huang et al., 2007). For the pathway analysis, 11 pathways were involved in the 40 up-regulated glycoproteins, including lysosome, metabolic pathways, glycan and glycosaminoglycan degradation, PI3K-Akt signaling pathways, etc. Seven pathways were involved in the 31 down-regulated glycoproteins, such as lysosome, cell adhesion molecules, extracellular matrix–receptor interaction, protein processing in the endoplasmic reticulum, etc. As the primarily involved pathway group, we discovered that the 11 up-regulated glycoproteins and the 7 down-regulated glycoproteins which participated in the lysosome pathway for protein degradation are displayed in S6A. The other two most involved pathways are metabolic pathway and glycan and glycosaminoglycan degradation pathway, which included 10 and 7 glycoproteins separately. Interestingly, for glycan and glycosaminoglycan degradation pathway, all these seven glycoproteins were up-regulated in *FUT8* KO CHO-K1 cells, and they all participated in N-glycan biosynthesis (Man2b1, Man2b2, Neu1, Glb1, Gba, and LOC100756951) and glycosphingolipid biosynthesis (Gns, Glb1, and LOC100756951) as glycosidase (Figures S6B, C).

## Conclusion

In summary, we developed and implemented a one-step method for the rapid and efficient enrichment of intact glycopeptides for mass spectrometry analysis. This method saved sample preparation time and improved the coverage of the glycopeptides for different glycosites. Furthermore, we characterized the differences in glycoprofiles between WT and *FUT8* knockout CHO-K1 cells and observed that the knockout of *FUT8* eliminates the core fucosylation in CHO-K1 cells and induces alterations of overall glycosylation. Moreover, we also identified changes in the type and the amount of N-linked glycopeptides from WT and *FUT8* knockout CHO-K1 cells, including the number of glycoproteins up-regulated and down-regulated that are involved in lysosome, metabolic, glycosylation, and PI3K-Akt signaling and other pathways. These findings indicated that future N-glycosylation profiling can be both enhanced in quality at the site-specific level and made more rapid by combining multi-column enrichment processes into a single column. More intriguingly, except for the site-specific glycosylation characterization, intact glycopeptide can also reveal the intracellular glycoprotein dynamics which is unachievable for the conventional glycan analysis methods and give insights for bio-analysts and biotechnologists to better tailor therapeutic drugs in the coming decades.

## Data Availability Statement

The raw data supporting the conclusions of this article will be made available by the authors, without undue reservation, to any qualified researcher.

## Author Contributions

HZ, GY, and S-YC conceived the study. GY designed and performed the IGP enrichment and MS analysis and conceived the bio-informatic analysis. NH and QW performed cell culture experiments. MB provided the Fut8 mutant cell lines. S-YC and YZ performed and supervised glycoproteomics experiments. GY, HZ, QW, and MB wrote and revised the manuscript.

## Conflict of Interest

The authors declare that the research was conducted in the absence of any commercial or financial relationships that could be construed as a potential conflict of interest.

## Abbreviations

IGP: intact glycopeptide
CHO: Chinese hamster ovary
MAX: mixed anion exchange
PSM: peptide spectrum match.
