# Supplementary material for: One-Step Enrichment of Intact Glycopeptides From Glycoengineered Chinese Hamster Ovary Cells
Source: Front Chem. 2020 Apr 17;8:240. doi: 10.3389/fchem.2020.00240 (PMC7180227; doi:10.3389/fchem.2020.00240)
Supplement: Supplementary file 6 [file Data_Sheet_1.DOCX]

**One-step enrichment of intact glycopeptides from glycoengineered Chinese hamster ovary cells**

Ganglong Yang^1^, Naseruddin Höti^1^, Shao-Yung Chen^1^, Yangying Zhou^1^, Qiong Wang^2^, Michael Betenbaugh^2^, Hui Zhang^1^

^1^Department of Pathology, Johns Hopkins University, Baltimore, Maryland 21231, USA

^2^Chemical and Molecular Engineering, Johns Hopkins University, Baltimore, Maryland 21218, USA

**Correspondence:** Dr. Hui Zhang, Department of Pathology, Johns Hopkins University, Baltimore, Maryland 21231, USA

**E-mail**: hzhang32@jhmi.edu

**Keywords:** Intact glycopeptides; Glycoengineered CHO cells; One-step enrichment; *FUT8* knockout; Mass spectrometry

**Abbreviations:** **IGP,** intact glycopeptide; **CHO,** Chinese hamster ovary; **MAX**, mixed anion exchange; **PSM**: peptide spectrum match

Figure S1


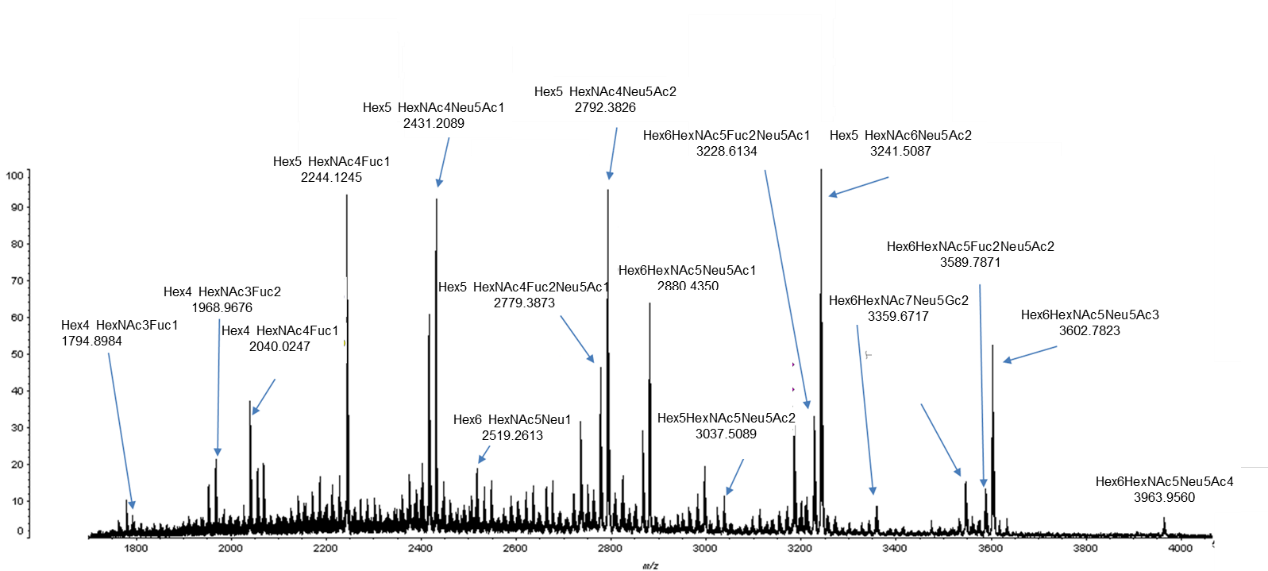


Figure S1: The MALDI spectra of permethylated N-linked glycans identified from Bovine Fetuin.

Figure S2


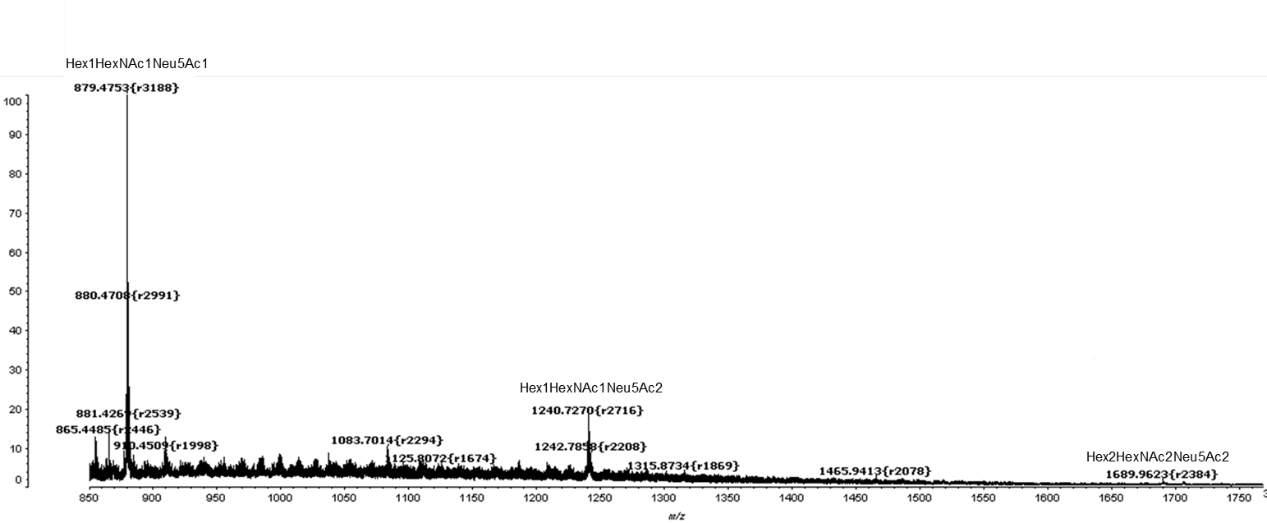


Figure S2: The MALDI spectra of permethylated O-linked glycans identified from Bovine Fetuin.

Figure S3


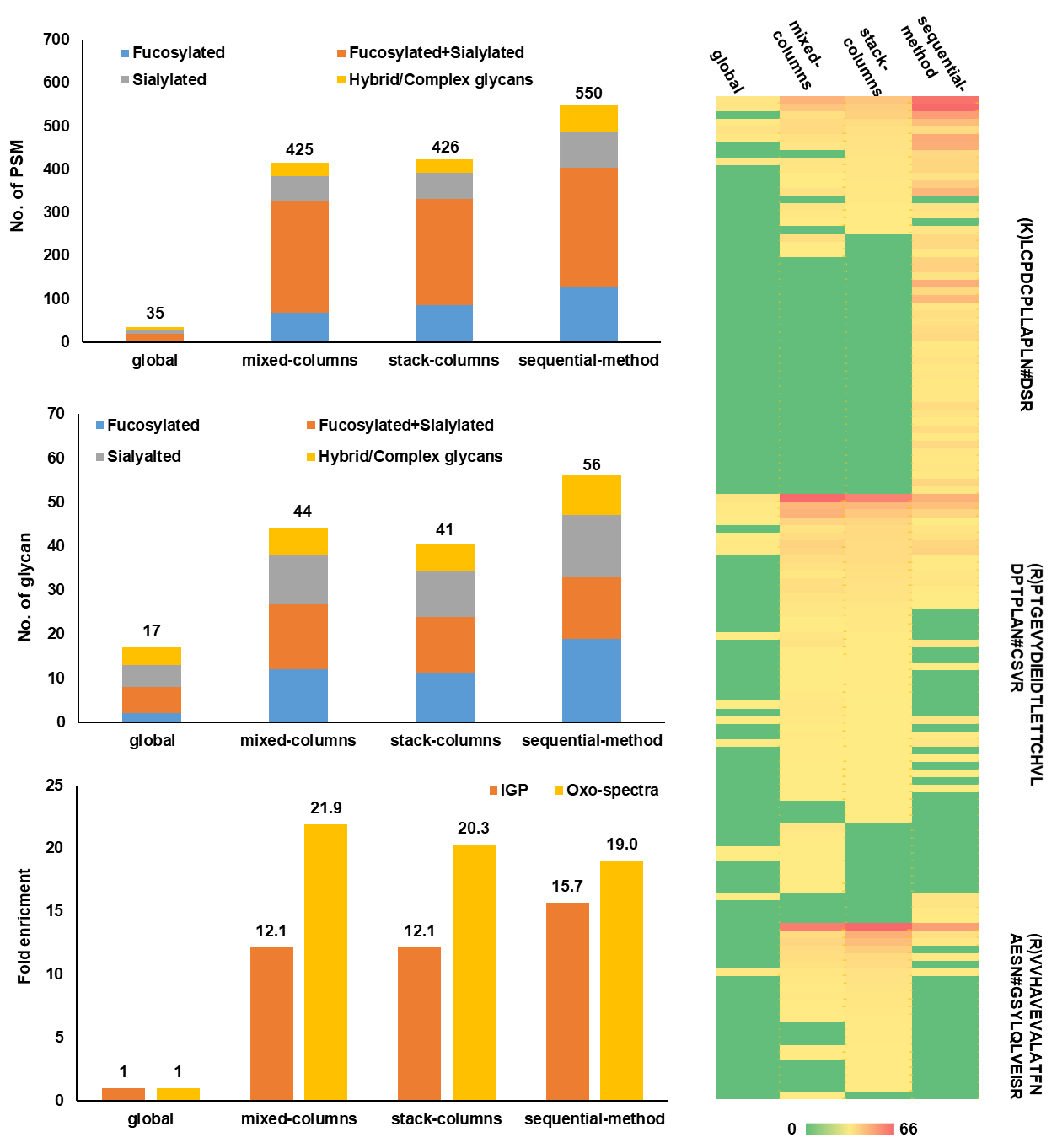


**Figure S3:** Enrichment of intact glycopeptides from Fetuin by one-step and sequenctial methods. The heatmap of site-specific glycosylation peptides distribution enriched by the one-step and seqeuntial methods.

Figure S4


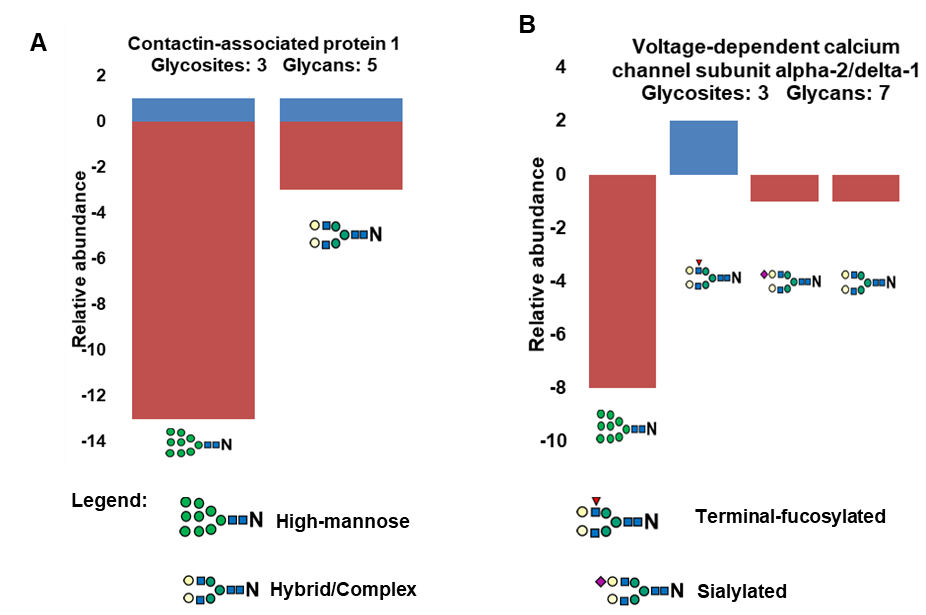


**Figure S4.** Glycosylation relative abundance of Contactin-associated protein 1 and Voltage-dependent calcium channel subunit alpha-2/delta-1 between the WT and *FUT8* KO CHO-K1 cells

Figure S5


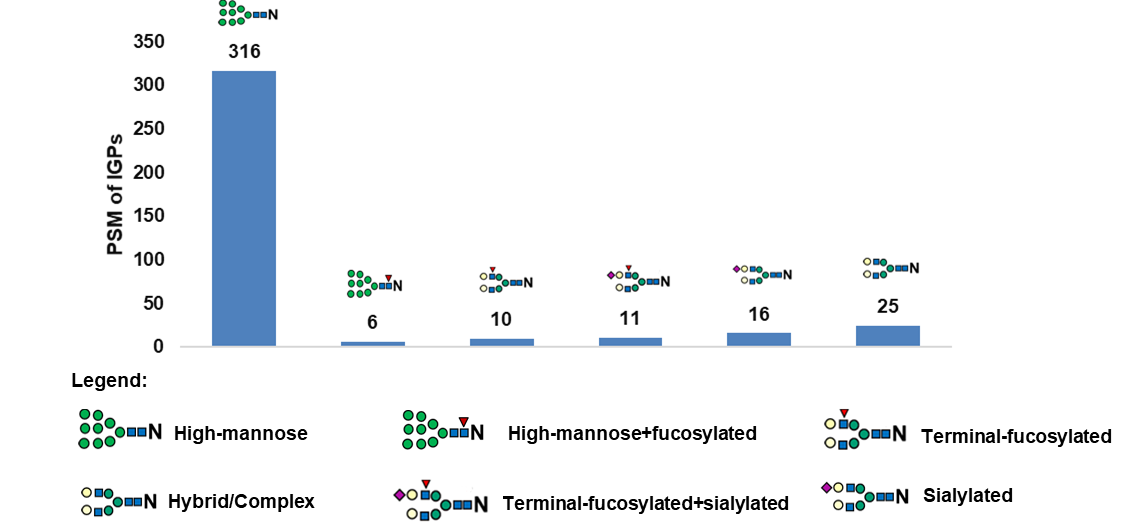


**Figure S5.** Glycosylation relative abundance of 90 proteins only identified in *FUT8* KO CHO-K1 cells

Figure S6


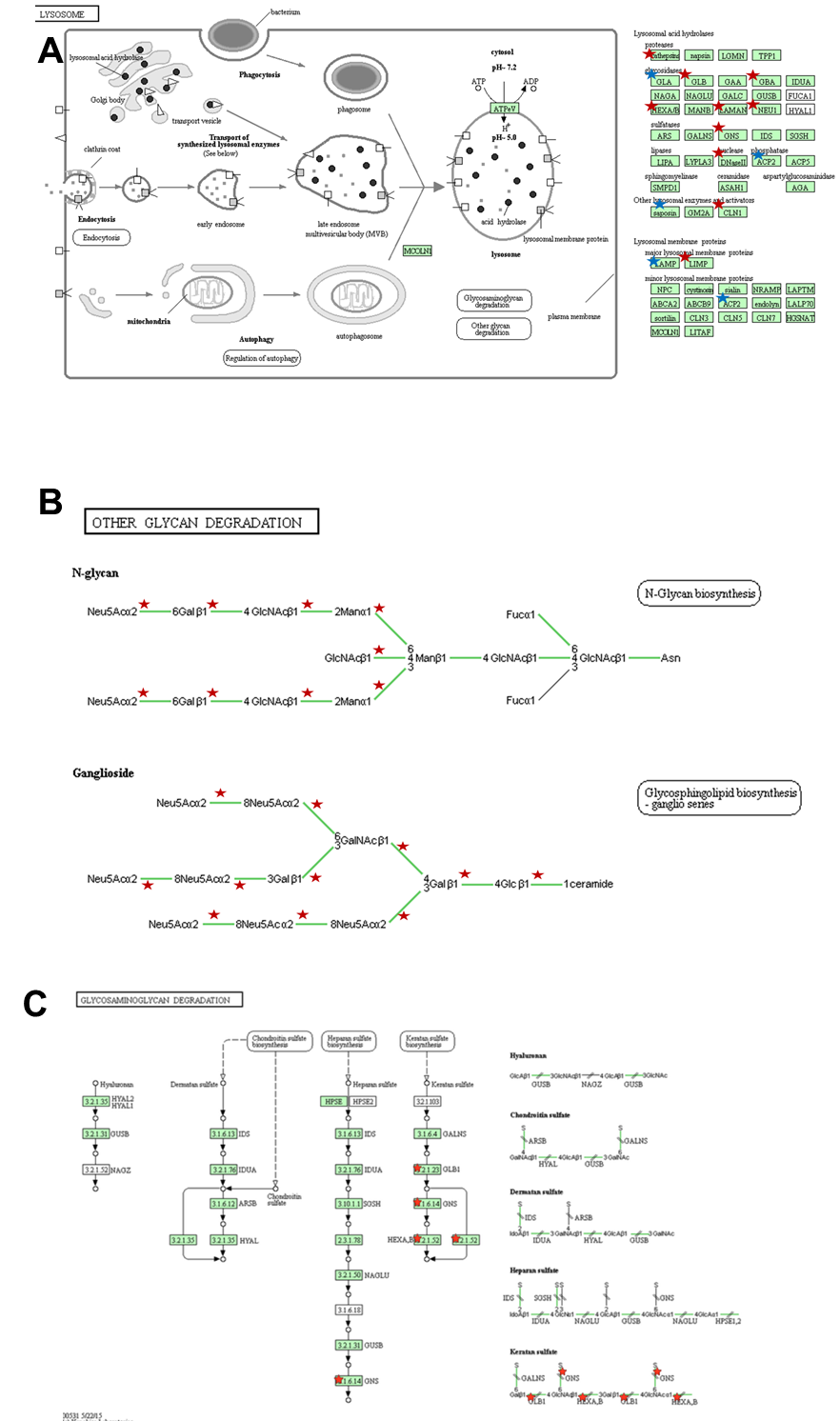


**Figure S6.** Pathway analysis of glycoproteins between the WT and *FUT8* KO CHO-K1 cells. The change of lysosome, glycan and glycosyaminoglycan degradation related pathway with the *FUT8-*KO cells. Red star: Up-regulated glycoproteins in the *FUT8* KO CHO cells. Blue star: Down-regulated glycoproteins in the *FUT8* KO CHO cells.
